# Supplementary material for: Phosphoproteomics identifies a bimodal EPHA2 receptor switch that promotes embryonic stem cell differentiation
Source: Nat Commun. 2020 Mar 13;11:1357. doi: 10.1038/s41467-020-15173-4 (PMC7070061; doi:10.1038/s41467-020-15173-4)
Supplement: Supplementary file 1 — Supplementary Information [file 41467_2020_15173_MOESM1_ESM.pdf]

Supplementary Information

**Supplementary Figures**	**p1**
**Supplementary Table 1: EPHA2 phosphopeptides identified from ESCs**	**p16**
**Supplementary Table 2: List of primary antibodies**	**p17**
**Supplementary Table 3: Primers used for qRT-PCR**	**p18**
**Supplementary Methods**	**p19**
**Supplementary References**	**p26**

Supplementary Figure 1 (Related to Fig. 1)

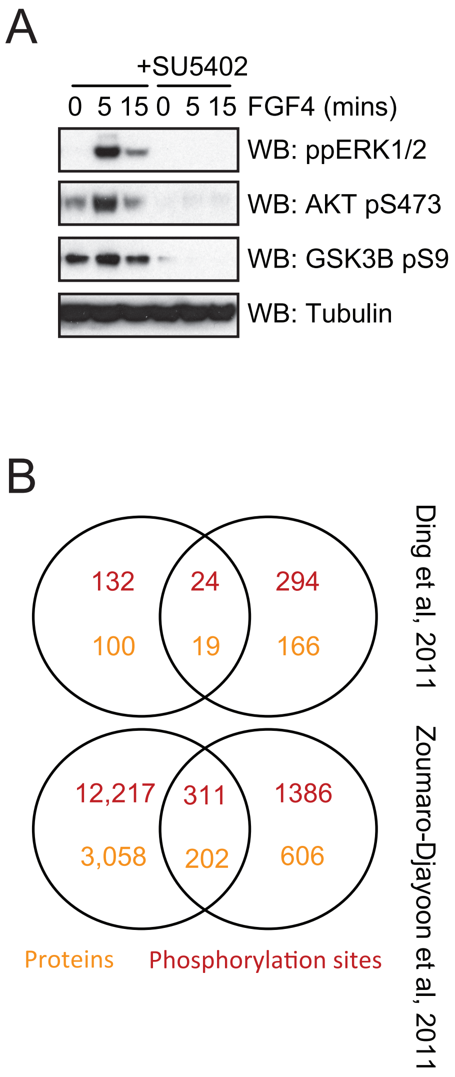

FGF4 phosphoproteomics analysis in mESCs.
A) *Fgf4*-/- mESCs were treated with 10 μM SU5402 for 1 h and stimulated with FGF4 for the indicated times. ppERK1/2, AKT pS473, GSK3B pS9 and tubulin levels were determined by immunoblotting. B) Venn diagrams showing the overlap between the phosphoproteomics data generated in this study and two previous large-scale phosphoproteomics datasets from FGF-stimulated ESCs. Source data are provided as a Source Data file.

Supplementary Figure 2 (Related to Fig. 2)

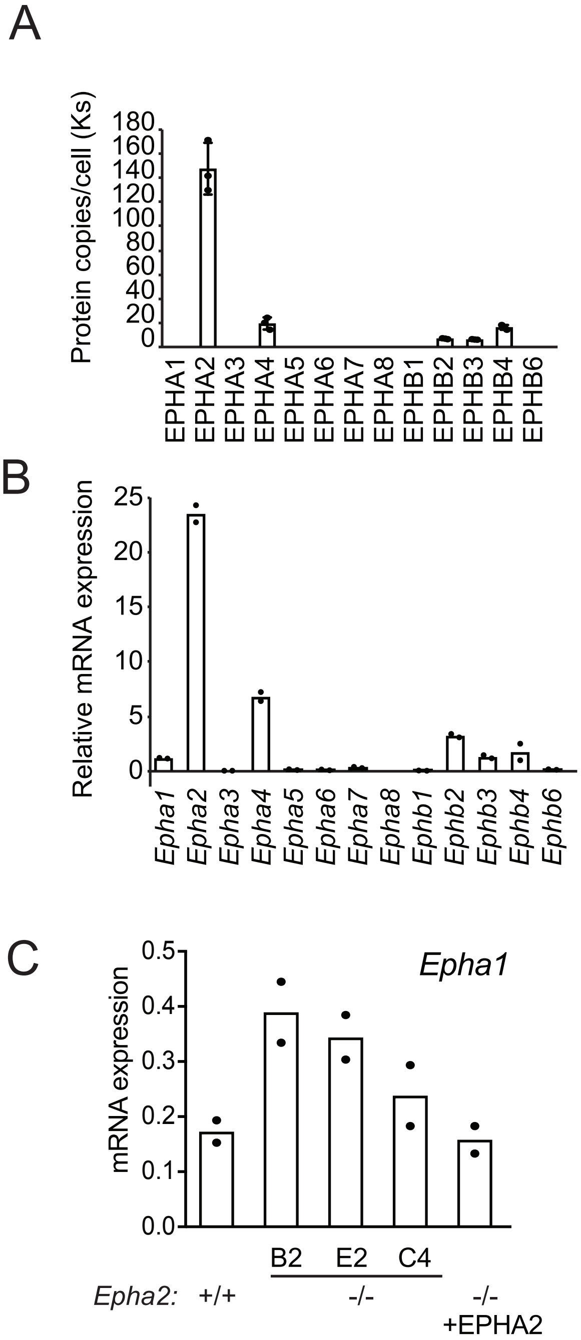

EPHA2 is the major EPH receptor expressed in mESCs.
A) Average protein copy number per cell for EPH receptor family members in mESCs using quantitative whole-cell proteomics. Data are presented as mean ± SD (n=3) B) mRNA expression of EPH receptor family members in mESCs determined by qRT-PCR analysis. Data presented as technical replicates of a representative experiment. C) mRNA expression of *Epha1* in *Epha2*+/+, *Epha2*-/- or *Epha2*-/- mESCs (clone C4) stably expressing EPHA2 was determined by qRT-PCR analysis. Data presented as technical replicates from a representative experiment. Source data are provided as a Source Data file.

Supplementary Figure 3 (Related to Fig. 3)

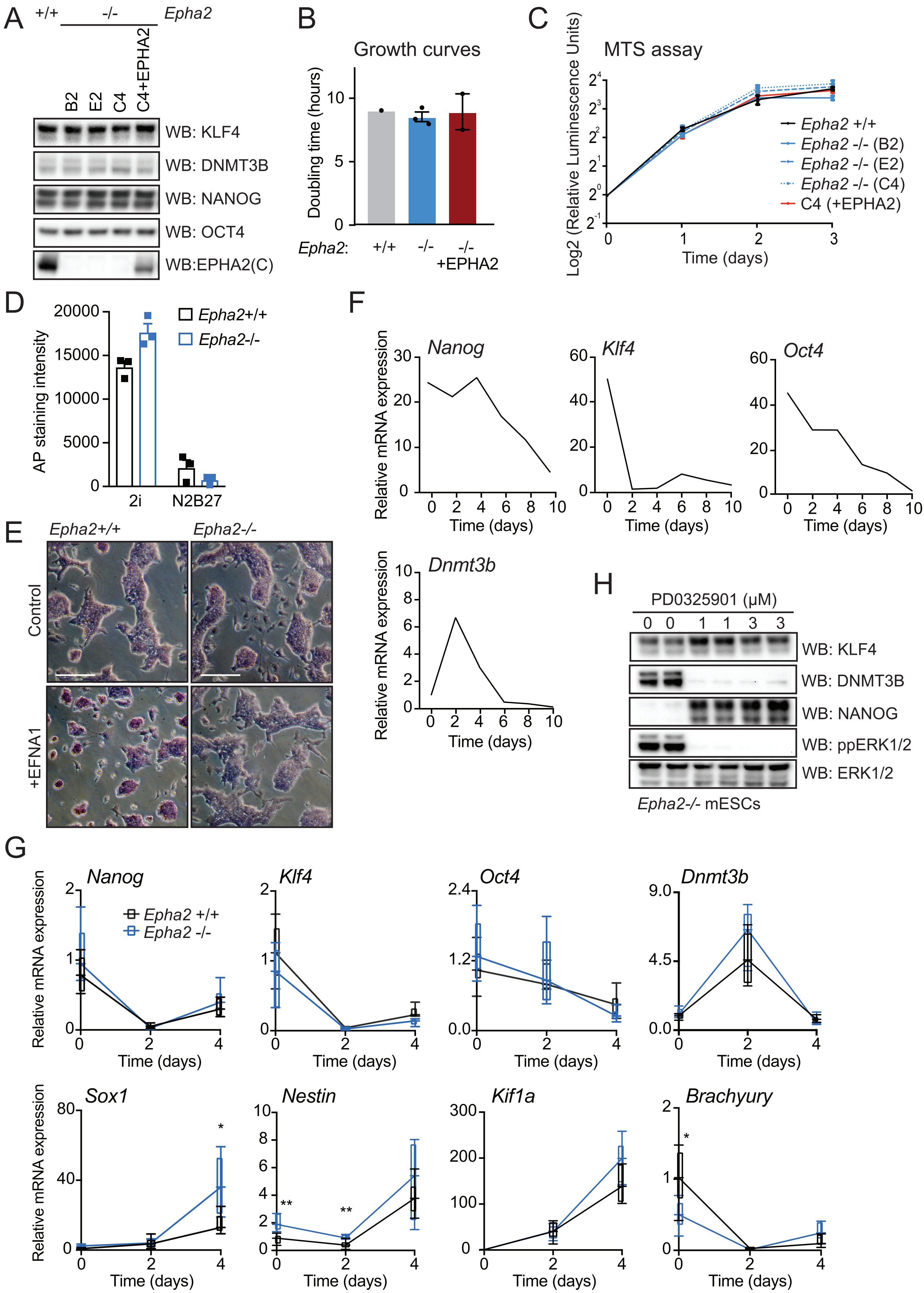

Supplementary Figure 3 (Continued)

EPHA2 supports mESC pluripotency and restricts commitment to differentiation.
A) *Epha2*+/+, *Epha2*-/- or *Epha2*-/- mESCs (clone C4) stably expressing EPHA2 were cultured in LIF/FBS for 48 h. EPHA2, KLF4, DNMT3B, NANOG and OCT4 levels determined by immunoblotting. B) *Epha2*+/+, *Epha2*-/- or *Epha2*-/- mESCs (clone C4) stably expressing EPHA2 were cultured in LIF/FBS and cells counted to determine doubling time. Data are represented as mean ± SEM (n=3). C) *Epha2*+/+, *Epha2*-/- or *Epha2*-/- mESCs (clone C4) stably expressing EPHA2 were cultured in LIF/FBS for 4 days. Viable cells were determined daily by MTS assay. Data are represented as mean ± SEM (n=3). D) Clonogenicity/density of alkaline phosphatase positive colonies was determined for *Epha2*+/+ or *Epha2*-/- mESCs (clone C4) in 2i or following 4 days differentiation in N2B27 media. Data show mean ± SEM (n=3). E) *Epha2*+/+ or *Epha2*-/- mESCs (clone C4) stably expressing EFNA1 were cultured for three days in N2B27 and replated in standard LIF/FBS medium for 24 h. Alkaline phosphatase activity was determined and bright field pictures taken; Scale bar, 100 μ m. F) Wild-type mESCs were differentiated as embryoid bodies for 10 days and levels of *Nanog*, *Klf4*, *Oct4* and *Dnmt3b* mRNA determined by qRT-PCR. Data are presented as mean of technical replicates from a representative experiment. G) *Epha2*+/+ or *Epha2*-/- mESCs (clone C4) cultured in 2i were differentiated in N2B27 media for 4 days and levels of *Nanog*, *Klf4*, *Oct4* and *Dnmt3b, Sox1, Nestin, Kif1a* and *Brachyury* mRNA determined by qRT-PCR. Box and whisker plots show median, first and third quartiles, and maximum and minimum values of two technical and three biological replicates (n=3). Statistical significance was determined using multiple unpaired t-tests comparing each group to control, using Holm-Sidak correction with alpha = 0.05 (*Sox1* *P = 0.013999, *Nestin* (0 days) **P = 0.001228, *Nestin* (4 days) **P = 0.001450, *Brachyury* *P = 0.014190) H) *Epha2*-/- clone C4 was cultured in the absence of LIF and treated with 1 or 3 μM PD0325901 (MEK1/2i) for 48 h. KLF4, DNMT3B, NANOG, ppERK1/2 and ERK1/2 levels were determined by immunoblotting. Source data are provided as a Source Data file.

Supplementary Figure 4 (Related to Fig. 4)

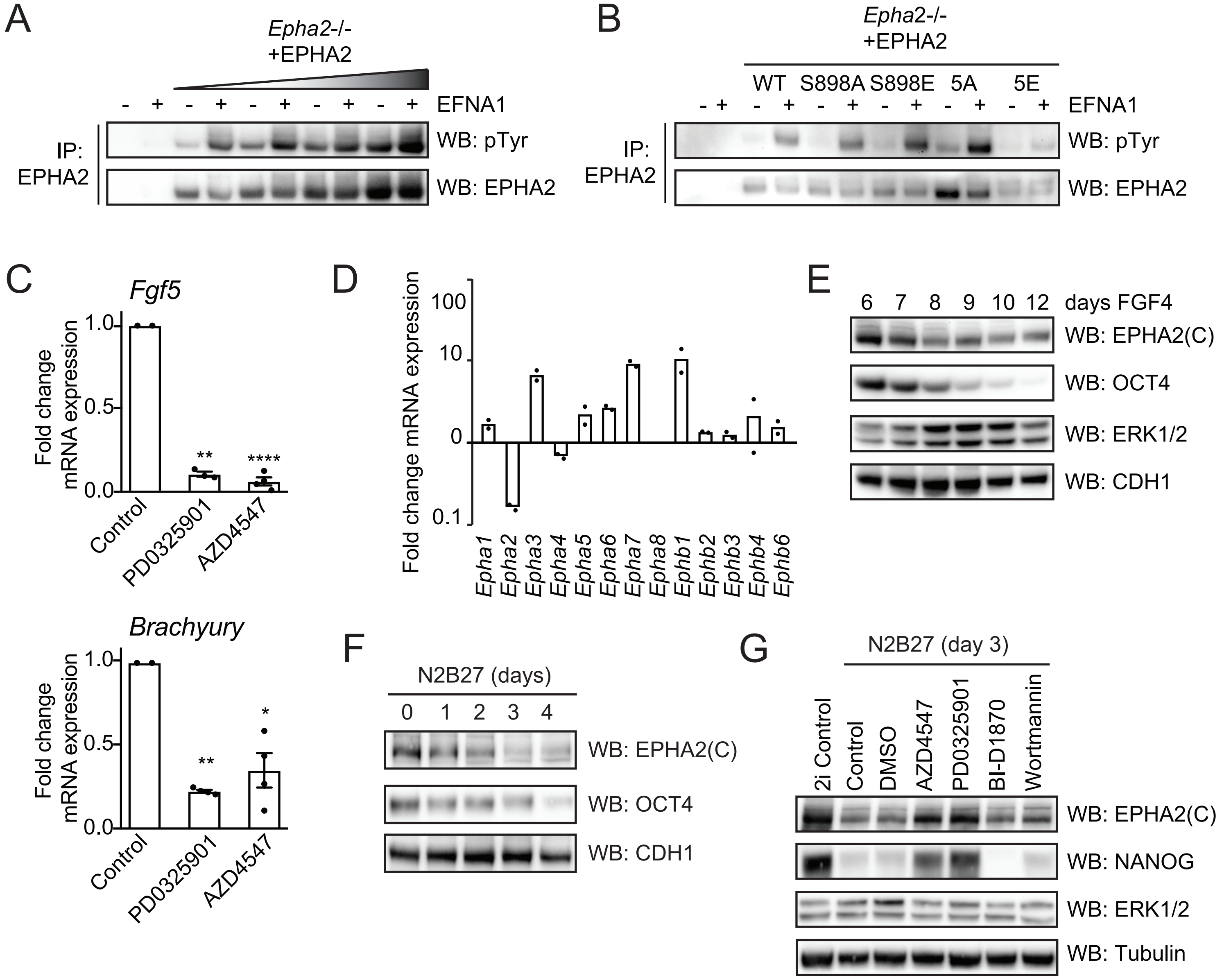

EPHA2 inhibitory phosphorylation and transcriptional suppression during mESC differentiation.

A) *Epha2*-/- mESCs were transfected with either empty vector or increasing amounts of EPHA2 and stimulated with 1 µg/ml clustered EFNA1 for 15 min. EPHA2 was immunoprecipitated and pTyr and EPHA2 levels determined by immunoblotting. B) *Epha2*-/- mESCs were transfected with either empty vector or indicated EPHA2 expression vectors and stimulated with 1 µg/ml clustered EFNA1 for 15 min. EPHA2 was immunoprecipitated and pTyr and EPHA2 levels determined by immunoblotting. C) *Epha2*+/+ mESCs were differentiated as embryoid bodies (EBs) for 10 days in the presence of vehicle control, 1 µM PD0325901 or AZD4547, and levels of *Fgf5* (top) and *Brachyury* (bottom) mRNA determined by qRT-PCR. Fold change in mRNA expression compared to control is presented as mean ± SD (n=4). statistical significance was determined using unpaired two-sided Student's t-test comparing each group to the control (*Fgf5* **P = 0.019, ****P < 0.0001, *Brachyury* **P = 0.0027, *P = 0.0211) D) EPH receptor mRNA expression was determined by qRT-PCR in mESCs or EBs differentiated for 6 days. Fold change of expression between EBs and mESCs is presented from two independent experiments. E) *Fgf4*-/- mESCs were differentiated in the presence FGF4 for the indicated times, and EPHA2, OCT4, ERK1/2 and CDH1 levels determined by immunoblotting. F) 2i mESCs were differentiated in N2B27 medium for the indicated times and EPHA2, OCT4, and CDH1 levels were determined by immunoblotting. G) 2i mESCs were treated with 2 µM of the indicated inhibitors and differentiated in N2B27 medium for 3 days. EPHA2, NANOG, ERK1/2 and tubulin levels were determined by immunoblotting. Source data are provided as a Source Data file.

Supplementary Figure 5 (Related to Fig. 5)

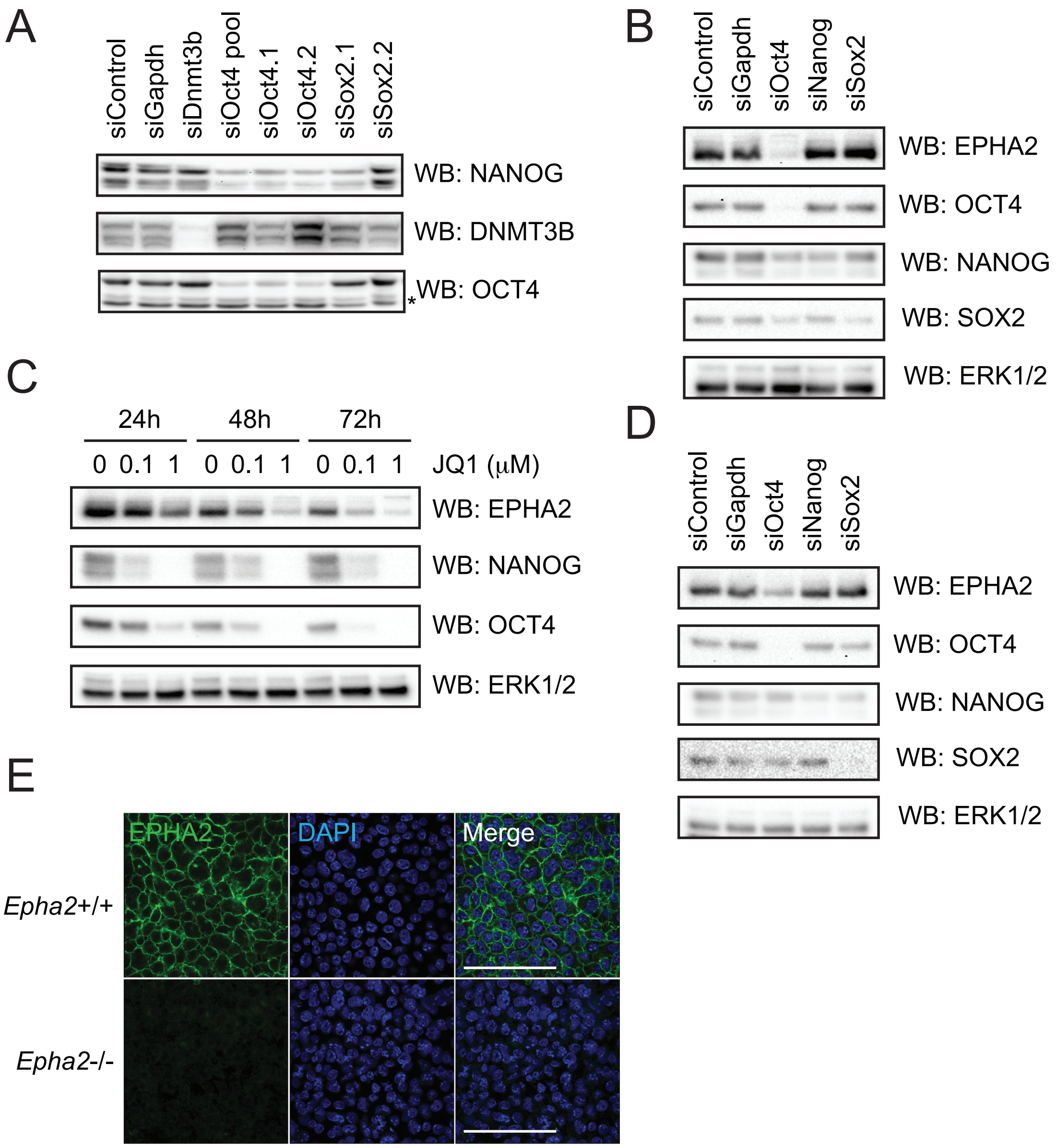

OCT4 controls EPHA2 expression in mESCs.

A) Wild-type mESCs were transfected with the indicated siRNAs and NANOG, DNMT3B and OCT4 levels determined by immunoblotting. (*) = non-specific band. B) Wild-type mESCs were transfected with the indicated siRNAs for 24h and EPHA2, OCT4, NANOG, SOX2 and ERK1/2 levels determined by immunoblotting. C) Wild-type mESCs were treated with JQ1 for the indicated time and concentration and EPHA2, NANOG, OCT4 and ERK1/2 levels determined by immunoblotting. D) Wild-type mESCs were transfected with the indicated siRNAs and EPHA2, OCT4, NANOG, SOX2 and ERK1/2 levels determined by immunoblotting. E) *Epha2+/+* and *Epha2-/-* mESCs were fixed, immunostained for EPHA2 and nucleus counterstained for DAPI. Scale bar = 50 µm. Source data are provided as a Source Data file.

Supplementary Figure 6 (Related to Fig. 6)

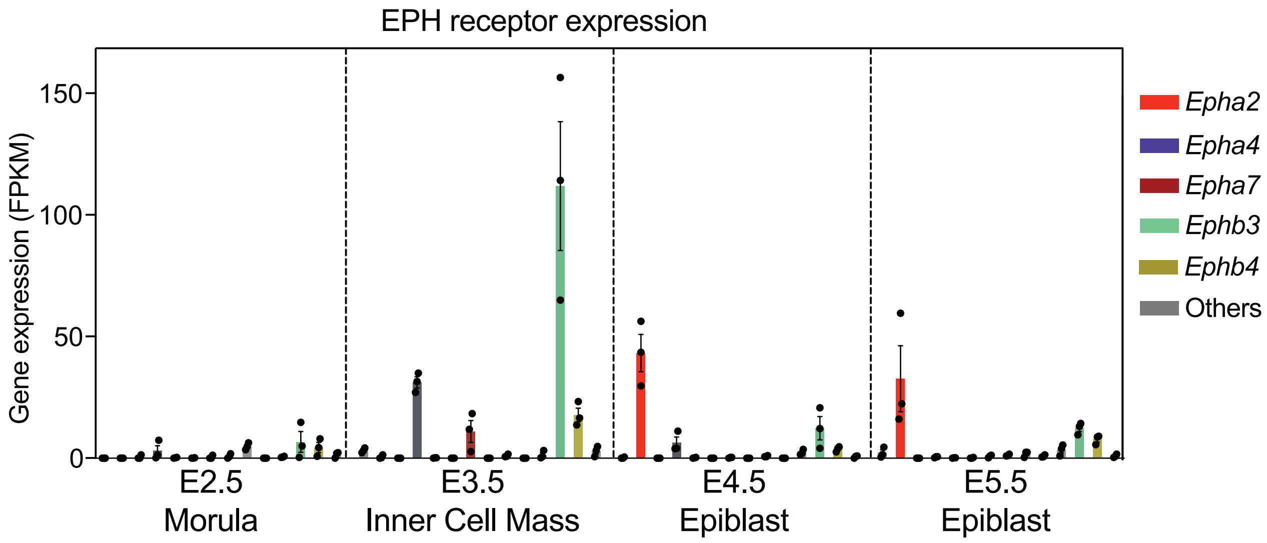

EPH receptor expression during a time-course of early mouse embryonic development

Data corresponding to EPH receptor mRNA expression was extracted from Table S1 in Boroviak *et al.*, 2015. Data are represented as mean ± SEM at each of four embryonic stages (n=3). Source data are provided as a Source Data file.

-

Supplementary Figure 7

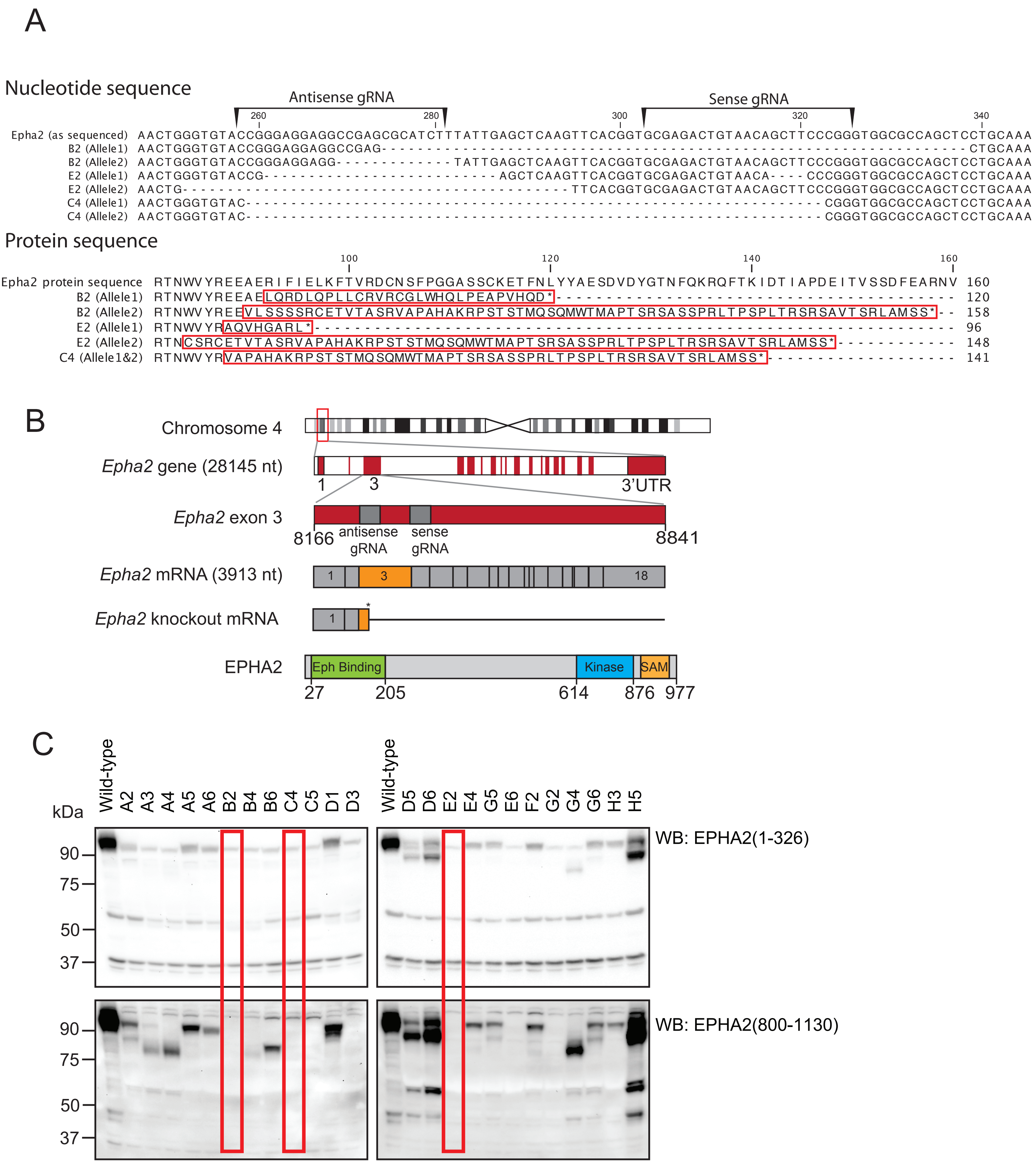

Generation of CRISPR/Cas9 *Epha2-/-* cell lines

A) *Epha2*-/- mESCs were generated using CRISPR/Cas9 D10A and the indicated sense and antisense gRNA sequences as described in experimental procedures. Genomic and predicted protein sequences of 3 independent *Epha2*-/- clones were determined. B) Schematic of the predicted *Epha2* gene and protein in *Epha2*-/- clones. C) Immunoblot analysis of EPHA2 expression using N-terminal and C-terminal specific antibodies in control wild-type mESCs and prospective *Epha2*-/- mESC clones. mESC lines that were selected for further analysis and experiments are indicated.

Supplementary Figure 8

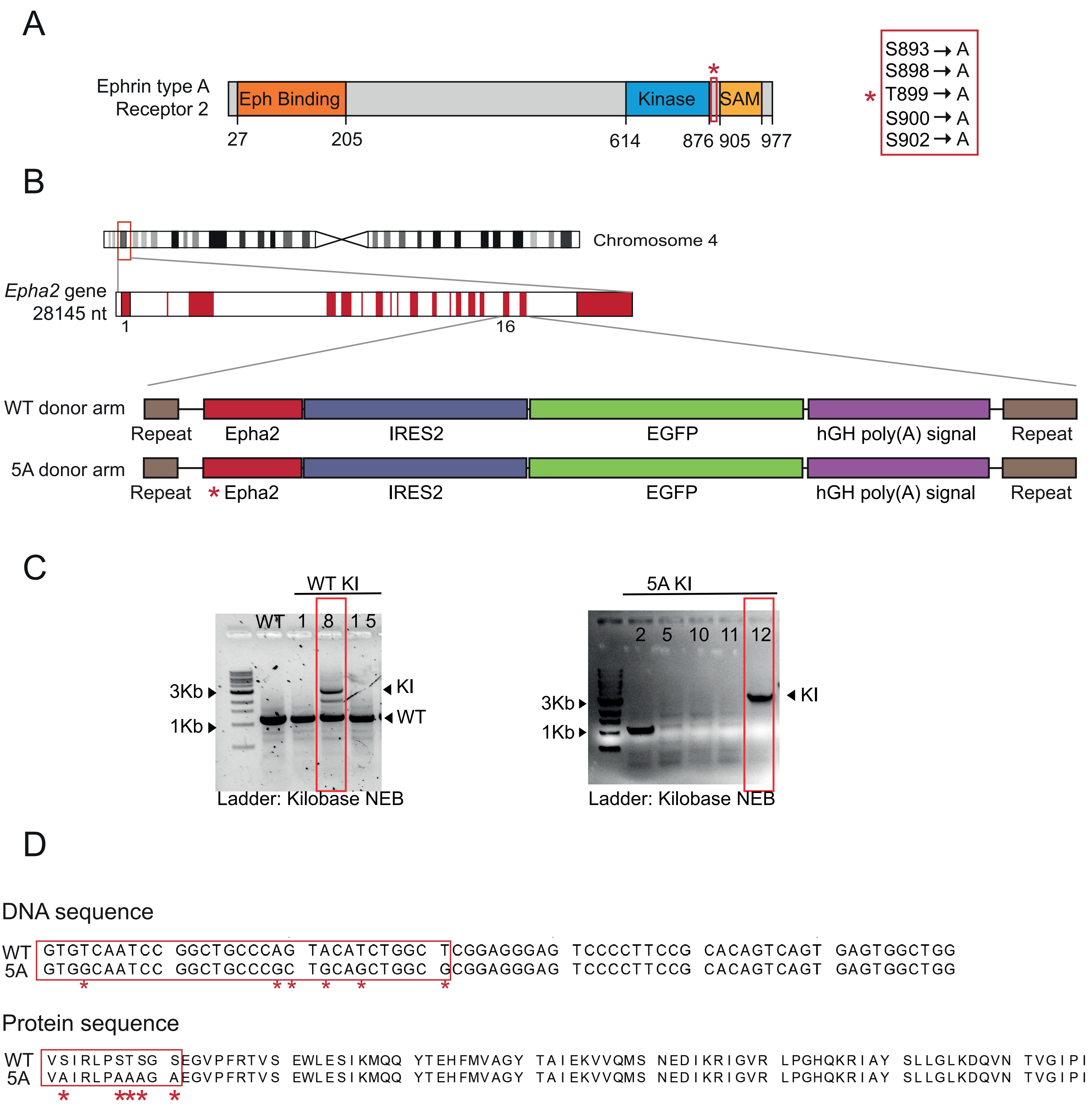

***Epha2* CRISPR/Cas9 knockin strategy**

A) CRISPR/Cas9 method was used to introduce 5 point mutations in the C-terminal region of EPHA2. B) Paired CRISPR/Cas9 guide RNAs were designed to target exon 16 of the *Epha2* gene in order to replace endogenous EPHA2 with either EPHA2 WT or EPHA2 5A mutant. Genomic PCR (C) and DNA sequencing (D) of *Epha2* WT-KI and 5A-KI mESCs confirmed the presence of wild-type or introduction of the 5A-KI donor. In the case of the WT KI, only one allele was targeted by the donor arm and therefore three bands are shown in the gel. Sequencing revealed a 45 bp deletion in the upstream intron 15-16, which does not affect protein expression or function.

Supplementary Figure 9: Full-size blot scans

Figure 1

F

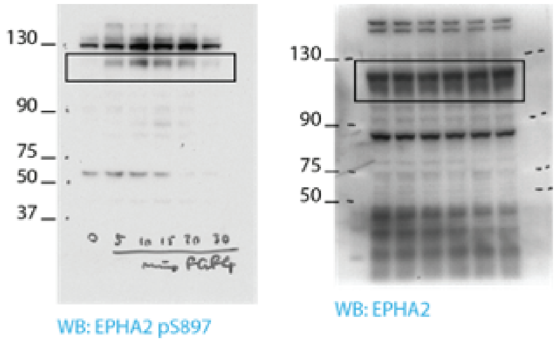

Figure 2

C

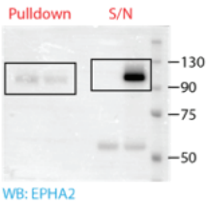

D

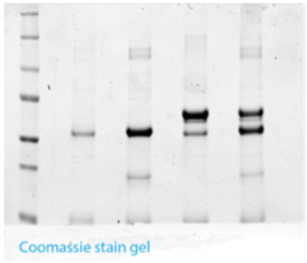

F

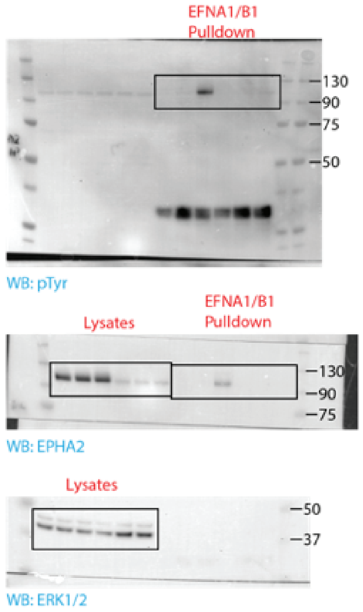

G

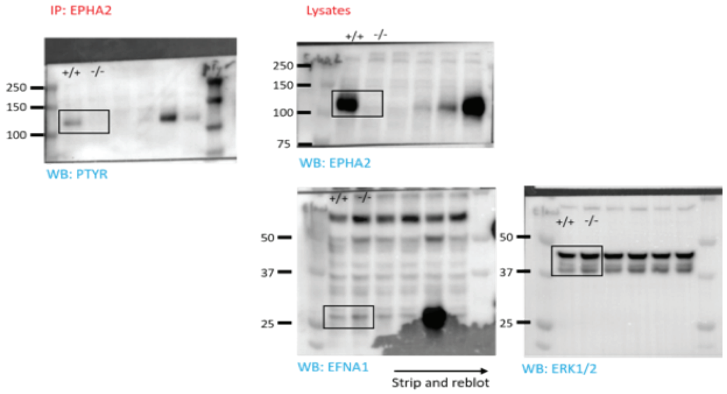

H

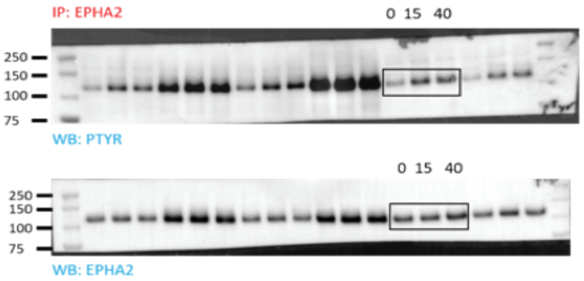

Figure 3

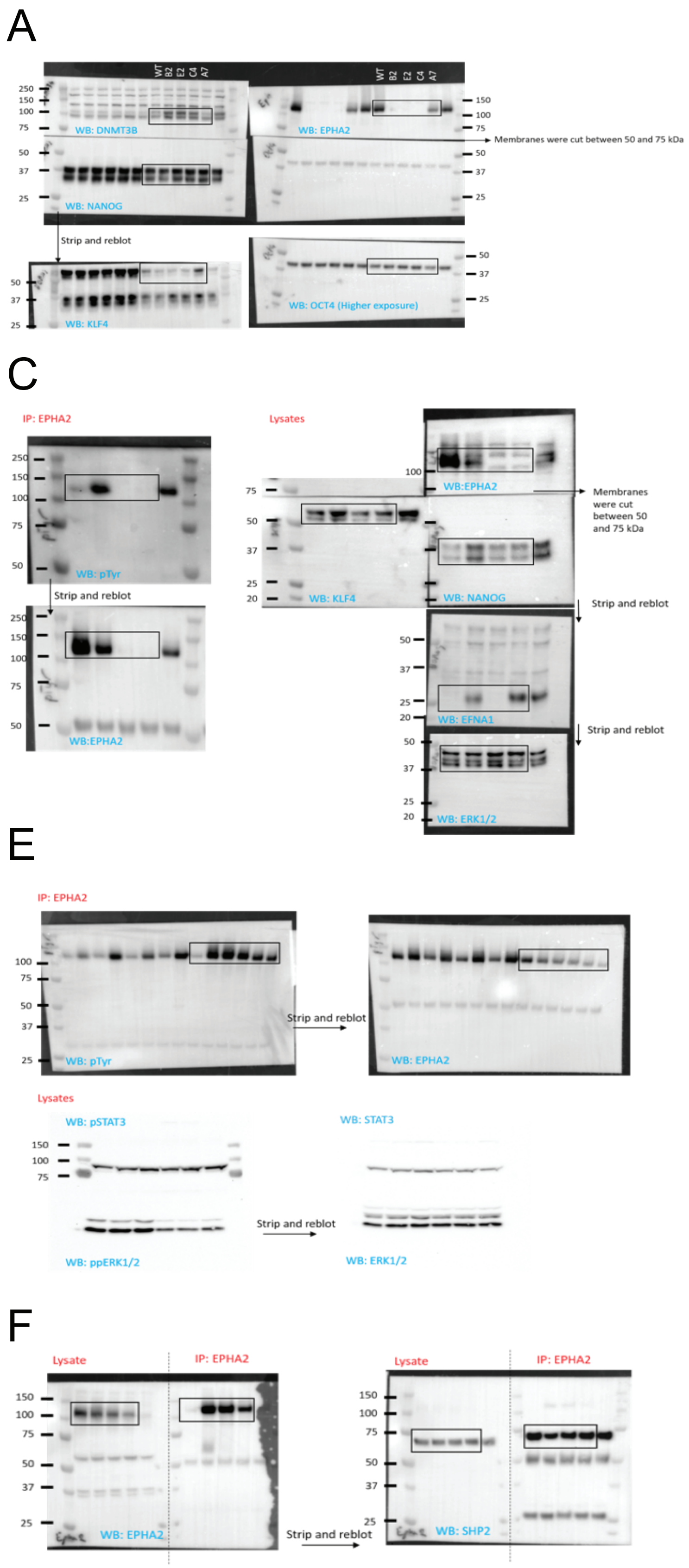
A
WT B2 E2 C4 A7
WB: DNMT3B
WB: EPHA2
Membranes were cut between 50 and 75 kDa
WB: NANOG
Strip and reblot
WB: KLF4
WB: OCT4 (Higher exposure)
C
IP: EPHA2
Lysates
WB: pTyr
Strip and reblot
WB:EPHA2
WB: KLF4
WB: NANOG
Membranes were cut between 50 and 75 kDa
Strip and reblot
WB: EFNA1
Strip and reblot
WB: ERK1/2
E
IP: EPHA2
WB: pTyr
Strip and reblot
WB: EPHA2
Lysates
WB: pSTAT3
WB: STAT3
Strip and reblot
WB: ppERK1/2
WB: ERK1/2
F
Lysate
IP: EPHA2
WB: EPHA2
Strip and reblot
Lysate
IP: EPHA2
WB: SHP2

Figure 4

B

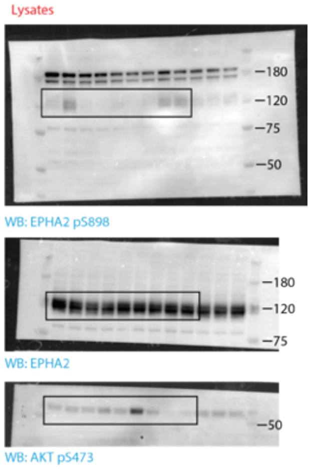

D

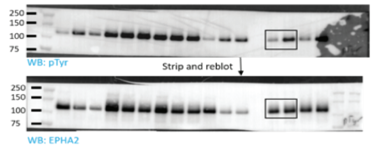

F

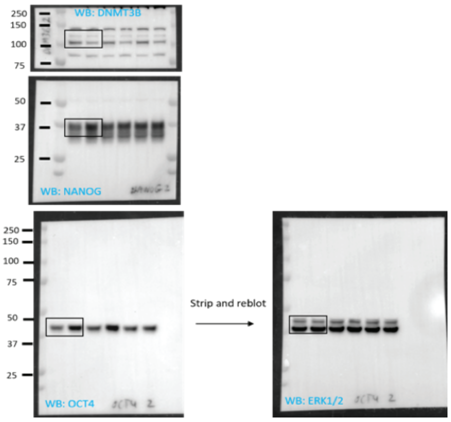

Figure 5

A

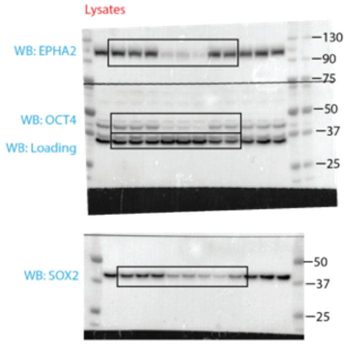

D

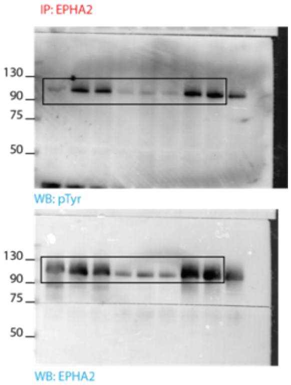

E

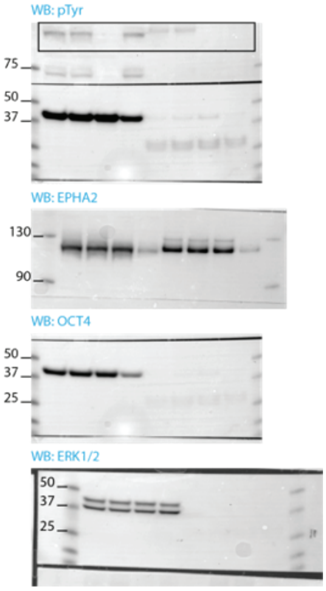

Supplementary Figure 1

A

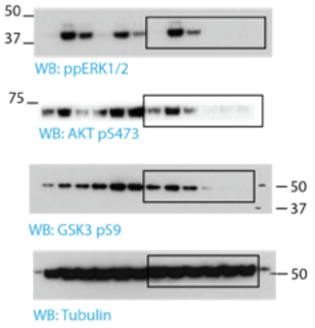

Supplementary Figure 3

A

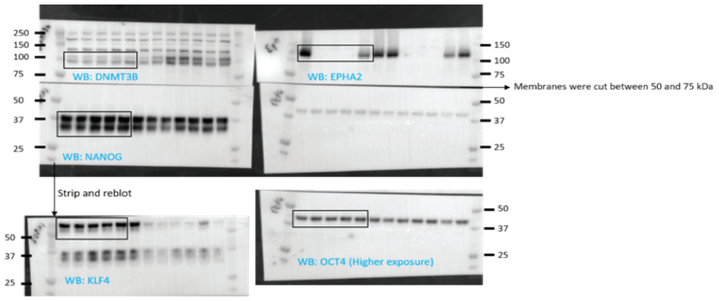

H

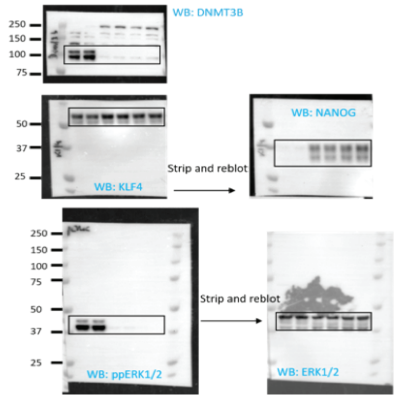

Supplementary Figure 4

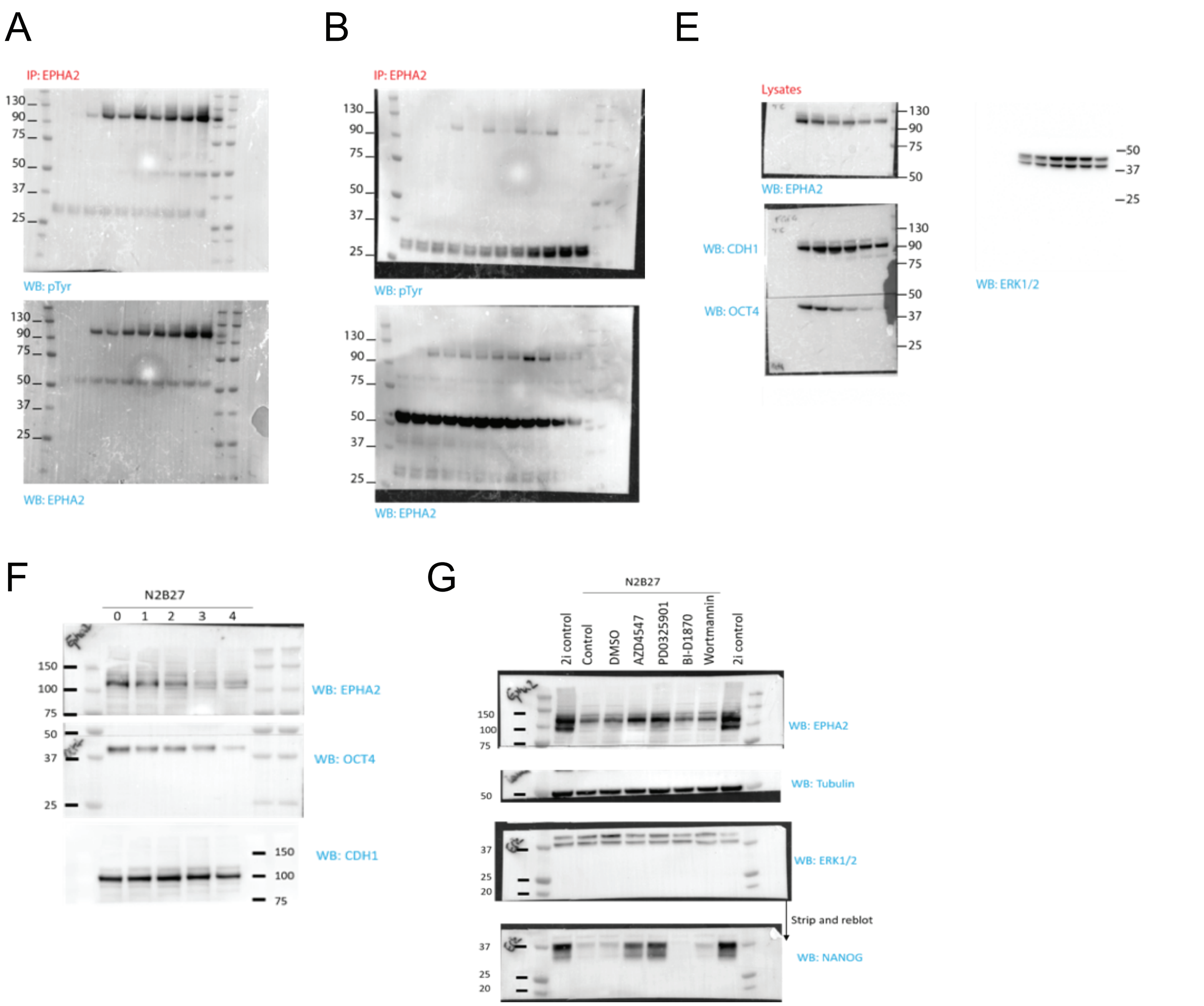

Supplementary Figure 5

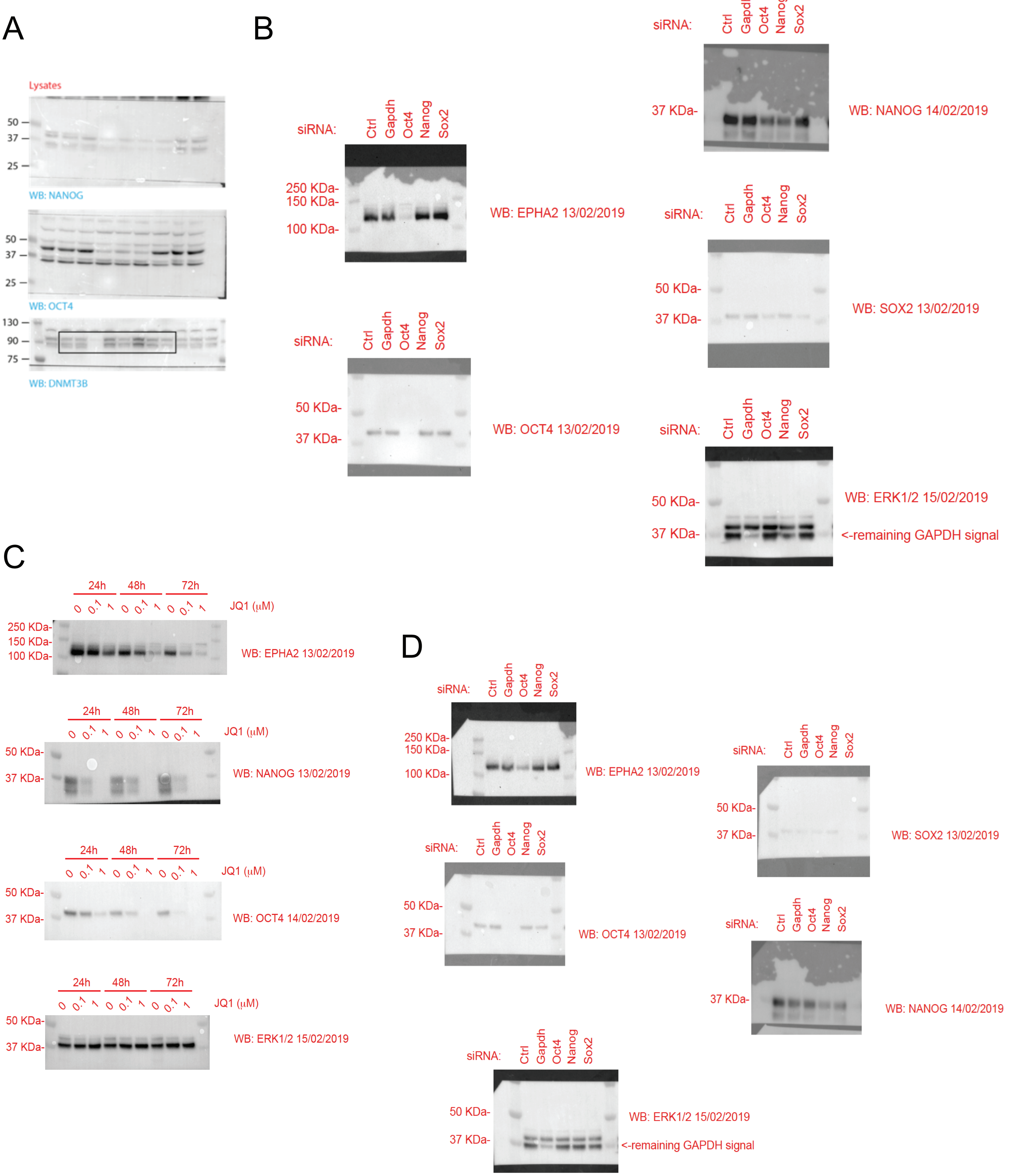

Supplementary Table 1: EPHA2 phosphopeptides identified from ESCs

GFindlay_141020_02

http://mascot.proteomics.dundee.ac.uk/cgi/master_results_2.pl?file=../data/20141106/F220797.dat

529.2876	1056.562	33	R.IAySLLGLK.D + Phospho (Y)
564.2441	1126.4768	21	R.ARQsSEDVR.F + Phospho (ST)
636.2976	1270.5846	26	R.TVsEWLESIK.M + Phospho (ST)
707.3231	1412.6337	45	R.LPsTSGSEGVPFR.T + Phospho (ST)
707.8215	1413.6323	39	K.VVQMsNEDIKR.I + Oxidation (M); Phospho (ST)
735.3594	1468.7075	46	K.TLADFDPRVsIR.L + Phospho (ST)
747.3049	1492.6	50	R.LPsTSGsEGVPFR.T + 2 Phospho (ST)
766.8235	1531.6456	20	R.tNWVYREEAER.I + Phospho (ST)
813.3799	1624.7498	20	R.APDsLKTLADFDPR.V + Phospho (ST)
583.2925	1746.8593	21	K.VIGAGEFGEVYKGtLK.A + Phospho (ST)
876.9388	1751.876	35	K.IPIRWtAPEAISYR.K + Phospho (ST)
881.8726	1761.7346	96	R.VLEDDPEATyTTSGGK.I + Phospho (ST)
921.8549	1841.7009	76	R.VLEDDPEAtyTTSGGK.I + Phospho (ST); Phospho (Y)
623.6459	1867.9193	54	R.VsIRLPSTSGSEGVPFR.T + Phospho (ST)
934.9655	1867.9193	35	R.VSIRLPSTSGSEGVPFR.T + Phospho (ST)
650.301	1947.8857	42	R.VSIRLPsTSGsEGVPFR.T + 2 Phospho (ST)
974.9487	1947.8857	43	R.VSIRLPSTSGSEGVPFR.T + 2 Phospho (ST)
1014.9288	2027.852	23	R.VsIRLPStSGsEGVPFR.T + 3 Phospho (ST)
676.9553	2027.852	22	R.VSIRLPSTSGSEGVPFR.T + 3 Phospho (ST)
690.6419	2068.9143	32	K.TYVDPHTYEDPNQAVLK.F + Phospho (Y)
1035.4608	2068.9143	71	K.TYVDPHTYEDPNQAVLK.F + Phospho (ST)
1047.5615	2093.1173	55	R.IAySLLGLKDQVNTVGIPI.- + Phospho (Y)
717.298	2148.8806	30	K.TYVDPHTYEDPNQAVLK.F + Phospho (ST); Phospho (Y)
1075.4454	2148.8806	71	K.TYVDPHTyEDPNQAVLK.F + Phospho (ST); Phospho (Y)
1108.0418	2214.0868	28	R.NILVNsNLVCKVSDFGLSR.V + Phospho (ST)
742.989	2225.9527	24	K.MQQyTEHFMVAGYTAIEK.V + Phospho (ST)
748.0231	2241.0566	42	R.VLEDDPEAtYTTSGGKIPIR.W + Phospho (ST)
1121.5317	2241.0566	68	R.VLEDDPEATYTTSGGKIPIR.W + Phospho (Y)
774.6793	2321.0229	37	R.VLEDDPEATYTTSGGKIPIR.W + Phospho (ST); Phospho (Y)
1161.5162	2321.0229	47	R.VLEDDPEAtYTTSGGKIPIR.W + Phospho (ST); Phospho (Y)
955.4479	2863.3307	25	K.TLADFDPRVsIRLPSTSGSEGVPFR.T + 2 Phospho (ST)
749.1188	2992.4583	35	K.SEQLKPLKTyVDPHTYEDPNQAVLK.F + Phospho (Y)
998.4917	2992.4583	28	K.SEQLKPLKTYVDPHTYEDPNQAVLK.F + Phospho (ST)
1025.1465	3072.4246	26	K.SEQLKPLKtYVDPHTYEDPNQAVLK.F + Phospho (ST); Phospho (Y)
769.1118	3072.4246	22	K.SEQLKPLKTYVDPHTYEDPNQAVLK.F + Phospho (ST); Phospho (Y)

Supplementary Table 2: List of primary antibodies

Immunoblotting	
ERK1/2 pT202/pY204	Cell Signaling Technology #9101
ERK1/2	Santa Cruz Biotechnology #sc-93
EPHA2 pS898	AbGent #AP3722a-ev
EPHA2 (N-TERMINAL)	MRC Reagents & Services S705D
EPHA2 (C-TERMINAL)	MRC Reagents & Services S700D
pTYR (CLONE 4G10)	Millipore #05-321
KLF4	R&D Systems #AF3158
DNMT3B	Imgenex #IMG-184A
NANOG	Reprocell #RCAB001P
OCT4	Santa Cruz Biotechnology #sc-5279
AKT pS473	Cell Signaling Technology #9271
GSK3B pS9	Cell Signaling Technology #9336
CDH1	BD Transduction Laboratories #610881
SOX2	Cell Signaling Technology #2748
EFNA1	R&D Systems #AF702
STAT3 pY705	Cell Signaling Technology #9131
STAT3α	Cell Signaling Technology #8768
SHP2	BD Biosciences #610621
α-TUBULIN	Sigma-Aldrich T9026
Immunostaining	
EPHA2	R&D Systems #AF639

Supplementary Table 3: Primers used for qRT-PCR

Mouse Gene	Forward	Reverse
Epha1	GACAGTTCCAGCCCTAGCAG	TCACCAGCTTCAGTGACAGG
Epha2	CTACCGCAAGAAGGGGGATG	GGAACTCGTGCACTTTGCTG
Epha3	CGGTGACAGTGACAGACCTC	GGTGATGGAGCAGCCTGATT
Epha4	AAGGCGTTGTCACCAAATGT	ATGACTGTAAAGCGGCCATC
Epha5	GGTGGGAAGAGATTGGTGAA	CCAACTGGTCAACAGCCAAT
Epha6	GGATGAACACAATAGGCCCATAC	TCCAGTTAGTACGAAGCCAGT
Epha7	AGGCTCTTCGCTGCTGTTAG	GCTGCAGTACTCGCTCCTTC
Epha8	CCTGTGAGCTGGGCTTCTAC	TCAGGTTCACTGGTGCTGAG
Ephb1	CGGAACCCAGCTAGTCTCAA	GTCATCCACGGTGGTAAAGG
Ephb2	AGAAGCCGAGTACCAGACCA	ACATACGATGGCAATGACGA
Ephb3	CATGGACACGAAATGGGTGAC	GCGGATAGGATTCATGGCTTCA
Ephb4	AGGTGCTGTCCACGTGTATG	CGTATCAGCTTCGCTCTCGT
Ephb6	ATTGCCTCAAGGTGAGCTGT	CAGTGATGGCTGCCAGTAGA
Efna1	GGAACAGTTCAAATCCCAAGTT	CTCGTAATGTGGGCAGATGAT
Efna2	TCAAGGTTTATGTGCGTCCA	TGCAGGAGCTGTTACTGGTG
Efna3	AACAGCTCCAATCAGCACCT	GAGCTGTTGTAGTGCGGACA
Efna4	CTACATCTCGGTGCCGACTC	CCCAACAGGATGAGCTGACT
Efna5	TTCATGATCGTGTTTTCGATG	GGCTGACTCATGTACGGTGT
Efnb1	GCCAAGCAAGGAGTCAGACA	TCCTGGTTCACAGTCTCATGC
Efnb2	CTTTGGAGGGCCTGGATAAC	CAGAACTTGCATCTTGTCCAACT
Efnb3	GCTTCTTGTCCTTCCTGTGC	GGGGAGCAGTTGGTATGAAA
Brachyury	TCCCGAGACCCAGTTCATAG	TTCTTTGGCATCAAGGAAGG
Fgf5	GCTGTGTCTCAGGGGATTGT	CACTCTCGGCCTGTCTTTTC
Mixl	GCTGCTACCCGAGTCCAGGAT	GCCTTGAGGATAAGGGCTGAAA
Cer1	GTCATCCTGCCCATCAAAAG	ATTTGCCAAAGCAAAGGTTG
Sox1	TTCCCCAGGACTCCGAGGCG	GCTGTGTGCCTCCTCTGCGG
Nestin	AACTCTCGCTTGCAGACACCTG	AGGTGCTGGTCCTCTGGTATCC
Kif1a	CACCACTATTGTCAACCCCAAA	CCCCAATGTCCCTGTAGACCT
Oct4	AGCTGCTGAAGCAGAAGAGG	AGATGGTGGTCTGGCTGAAC
Klf4	ACACTTGTGACTATGCAGGCTGTG	TCCCAGTCACAGTGGTAAGGTTTC
Nanog	CTCATCAATGCCTGCAGTTTTTCA	CTCCTCAGGGCCCTTGTCAGC
Dnmt3b	CTGGCACCCTCTTCTTCATT	ATCCATAGTGCCTTGGGACC
GAPDH	CTCGTCCCGTAGACAAAA	TGAATTTGCCGTGAGTGG

Supplementary methods

Phosphoproteomic profiling

Cell culture and proteomic sample preparation: Fgf4-/- mESCs were cultured in LIF/FBS then starved for 4 h before stimulation with 100 ng/ml FGF4 (Peprotech). Cell pellets were lysed with 1 ml of lysis buffer (8 M Urea, 50 mM Ammonium bicarbonate containing protease inhibitor and phosphatase inhibitor). Samples were left on ice for 15 min and sonicated for 5 min at high intensity (30 sec on and off) in ice-cold water bath. After sonication, benzonase® endonuclease (Merck Millipore) was added to the lysate (1:100) to degrade DNA and RNA[1]. The lysates were then centrifuged at 12,000 rpm for 15 min at 4 °C, and supernatants were transferred into new eppendorf tubes. Protein concentrations were measured by BCA assay (Pierce™ BCA Protein Assay Kit, Thermo). A total of 7.5 mg protein from each sample was reduced with 5 mM DTT at 45 °C for 30 min. Lysates were briefly centrifuged and cooled to room temperature before alkylation in the dark with 10 mM iodoacetamide at room temperature. The alkylation was quenched by the addition of 5 mM DTT. Samples were first digested by Lys-C at weight ratio of 1:200 (w/w) for 4 h at 30 °c. Samples were then diluted with 50 mM ammonium bicarbonate to 1.5 M Urea concentration, followed by trypsin (Pierce trypsin, Thermo) at an enzyme to protein ratio of 1:50 (w/w) at room temperature with gentle shaking for overnight. The digest was stopped by the addition of 1% (v/v) TFA, and samples centrifuged at 10,000 g for 10 min at room temperature. Supernatants were desalted on 200 mg SepPak tC18 cartridge (Waters), and desalted peptides dried by vacuum centrifugation using Speedvac (Thermo).

Phosphopeptide Enrichment and TMT labelling: All samples were enriched for phosphopeptides using a TiO_2 microspheres (Titansphere® TiO 5 µM, GL Science) according to[2-4]. In brief, TiO_2 microspheres were equilibrated with 50 % ACN/ 2 M lactic acid (binding buffer) twice. 5 mg of desalted sample was

dissolved in 1 ml of binding buffer and incubated with 1 mg of TiO_2 microspheres with high speed shaking (1400 rpm) for 1 h at room temperature. TiO_2 microspheres were then washed twice with 1 ml binding buffer and three times with 1 ml of 50% ACN/0.1% TFA. After the final wash, TiO_2 microspheres were resuspended into 200 µl of 50% ACN/0.1% TFA and transferred to C8 Stage tip by centrifugation. The enriched phosphopeptides were then eluted twice with 200 µl 10% ammonium hydroxide. 50 µl of 50% ACN/5% ammonium hydroxide was used for final elution. All elutions were combined, acidified with TFA to a final concentration of 1% TFA, dried and desalted on an in-house C18 Ziptip 200 µl pipette tip. A small aliquot of enriched peptides were analysed by LC-MS/MS before TMT labelling.

TMT 9-plex reagents (0.8 mg, Thermo Fisher Scientific) were resuspended in 41 µl of anhydrous acetonitrile and added to the enriched phosphopeptides dissolved in 100 µl of 100 mM TEAB buffer. After 1 h incubation, the TMT labelling reaction was quenched by adding 8 µl of 5% hydroxylamine. An aliquot of labelled peptides was evaluated for labelling efficiency by LC-MS/MS prior to mixing. TMT labelled peptides were mixed, acidified, dried and then desalted by a 50 mg SepPak tC18 cartridge (Waters).

Basic C18 reverse phase (bRP) chromatography fractionation: An ultimate 3000 high-pressure liquid chromatography (HPLC) system (Dionex) was used for basic C18 reverse phase chromatography fractionation operating at 569 µl/min with two buffers: buffer A (10 mM ammonium formate, pH 10) and buffer B (80% ACN, 10 mM ammonium formate, pH 10). The desalted mixture of TMT-labelled peptides was resuspended in 200 µl of buffer A (10 mM ammonium formate, pH10) and separated on a C18 reverse phase column (4.6 × 250 mm, 3.5 µm, Waters) with a gradient from 3% B to 12.5 % B in 4.5 min, 12.5% to 60% buffer B in 42.5 min, 60% B to 90% B in 7.5 min, 90% for 2.5 min, ramping to

3% in 0.5 min and then 3% for 5 min. A total of 60 fractions (1 min per fraction) were collected before further concatenation into 20 final fractions as in[5]. Each fraction was then dried and desalted over a C18 StageTip prior to analysis by mass spectrometry.

LC-MS/MS analysis: The LC separations were performed with a Thermo Dionex Ultimate 3000 RSLC Nano liquid chromatography instrument. Approximately 1 µg of concatenated peptides (Peptides quantitation by Nanodrop) from bRP chromatography were dissolved in 0.1% formic acid and then loaded on C18 trap column with 3 % ACN/0.1%TFA at a flow rate of 5 µl/min. Peptide separations were performed over EASY-Spray column (C18, 2 µm, 75 µm × 50 cm) with an integrated nano electrospray emitter at a flow rate of 300 nl/min. Peptides were separated with a 180 min segmented gradient as follows: the first 7 fractions starting from 7%~25% buffer B in 125 min (Note: the middle 7 fractions starting from 10% and the last 6 fractions starting from 12.5%), 25%~45% buffer B in 30 min, 45%~95% buffer B for 5 min, followed by a 5 min 95% B. Eluted peptides were analysed on an Orbitrap Fusion Lumos (Thermo Fisher Scientific, San Jose, CA) mass spectrometer. Spray voltage was set to 2 kV, RF lens level was set at 30%, and ion transfer tube temperature was set to 275 °C. Full-scan resolution was set to 120 K with maximum ion injection time of 50 ms and automated gain control (AGC) target of 4 × 105. The mass spectrometer was operated in data-dependent top speed mode at 3 seconds per cycle. The survey scan was acquired from m/z 350 to 1500 with 120,000 resolving power and AGC target 4 × 105. The maximum injection time for full scan was set to 50 ms. The MS/MS analyses were performed by 1.2 Da isolation with the quadrupole, normalised HCD collision energy of 38% and analysis of fragment ions in the Orbitrap using 50,000 resolving power with auto normal range scan starting from m/z 100 and AGC target of 5 × 104. Dynamic exclusion was

set to 60 seconds. Monoisotopic precursor selection was set to peptide, maximum injection time was set to 120 msec. Charge states between 2 to 7 were included for MS2 fragmentation.

Data Analysis: All the acquired LC-MS data were analysed using Proteome Discoverer software v.2.2 (Thermo Fisher Scientific) with Mascot search engine. A maximum missed cleavages for trypsin digestion was set to 2. Precursor mass tolerance was set to 20 ppm. Fragment ion tolerance was set to 0.05 Da. Carbamidomethylation on cysteine (+57.021 Da) and TMT-10plex tags on N termini as well as lysine (+229.163 Da) were set as static modifications. Variable modifications were set as oxidation on methionine (+15.995 Da) and phosphorylation on serine, threonine, and tyrosine (+79.966 Da). Data were searched against a complete UniProt mouse (17,000 entries downloaded as of July 2018) with a 1% FDR using Percolator.

The phosphoRS[6] node was used to localize phosphorylation sites with a probability at least 90% to be considered as confident. Both unique and razor peptides were used for quantitation. Reporter ion abundances were corrected for isotopic impurities based on the manufacturer's data sheets. Signal-to-noise (S/N) values were used to represent the reporter ion abundance with a co-isolation threshold of 50% and an average reporter S/N threshold of 10 and above required for quantitation from each MS2 spectra to be used. The S/N value of each reporter ion from each PSM were used to represent the abundance of the localised phosphorylation sites. The precursor spectra with higher than 25% co-isolation were checked manually. The median was used for the normalisation and quantification calculated as average of three biological replicates, with standard deviation of three biological replicates lower than 25% used for further analyses.

Quantitative total cell proteomics

Sample preparation and TMT labelling: Cell pellets were lysed in 400 µL lysis buffer (4% SDS, 50 mM TEAB pH 8.5, 10 mM TCEP), boiled and sonicated with a BioRuptor (30 cycles: 30 sec on, 30 sec off) before alkylation with iodoacetamide for 1 h at room temperature in the dark. Lysates were subjected to the SP3 procedure for protein clean-up before elution into digest buffer (0.1% SDS, 50 mM TEAB pH 8.5, 1mM $CaCl_2$) and digest with LysC and Trypsin at a 1:50 (enzyme:protein) ratio. TMT labelling and peptide clean-up were performed according to the SP3 protocol. Samples were eluted into 2% DMSO, combined and dried in vacuo.

Basic reverse-phase fractionation: TMT samples were fractionated using off-line high pH reverse phase chromatography: samples were loaded onto a 4.6 x 250 mm Xbridge™ BEH130 C18 column with 3.5 µm particles (Waters). Using a Dionex BioRS system, the samples were separated using a 25-minute multistep gradient of solvents A (10 mM formate at pH 9 in 2% acetonitrile) and B (10 mM ammonium formate pH 9 in 80% acetonitrile), at a flow rate of 1 mL/min. Peptides were separated into 48 fractions, consolidated into 24, dried and peptides redissolved in 5% formic acid and analysed by LC-MS.

Liquid chromatography electrospray tandem mass spectrometry analysis (LC-ES-MS/MS): 1 µg of protein per fraction was analysed using an Orbitrap Fusion Tribrid mass spectrometer (Thermo Scientific) equipped with a Dionex ultra high-pressure liquid chromatography system (nano RSLC). RP-LC was performed using a Dionex RSLC nano HPLC (Thermo Scientific). Peptides were injected onto a 75 µm × 2 cm PepMap-C18 pre-column and resolved on a 75 µm × 50 cm RP- C18 EASY-Spray temperature controlled integrated column-emitter (Thermo) using a four hour multistep gradient from 5% B to 35% B with a constant flow of 200 nL min^{-1}. The mobile phases were: 2% ACN

incorporating 0.1% FA (Solvent A) and 80% ACN incorporating 0.1% FA (Solvent B). The spray was initiated by applying 2.5 kV to the EASY-Spray emitter and the data were acquired under the control of Xcalibur software in a data dependent mode using top speed and 4 s duration per cycle, the survey scan is acquired in the Orbitrap covering the *m/z* range from 400 to 1400 Th with a mass resolution of 120,000 and an automatic gain control (AGC) target of 2.0 e5 ions. The most intense ions were selected for fragmentation using CID in the ion trap with 30 % CID collision energy and an isolation window of 1.6 Th. The AGC target was set to 1.0 e4 with a maximum injection time of 70 ms and a dynamic exclusion of 80 s. During the MS3 analysis for more accurate TMT quantifications, 10 fragment ions were co-isolated using synchronous precursor selection using a window of 2 Th and further fragmented using HCD collision energy of 55%. The fragments were then analysed in the Orbitrap with a resolution of 60,000. The AGC target was set to 1.0 e5 and the maximum injection time was set to 300 ms.

Database searching and reporter ion quantification: The data were processed, searched and quantified with the MaxQuant software package, version 1.5.3.30. Proteins and peptides were identified using the UniProt *mouse* reference proteome database (SwissProt and Trembl accessed on 24.03.2016) and the contaminants database integrated in MaxQuant using the Andromeda search engine with the following search parameters: carbamidomethylation of cysteine and TMT modification on peptide N-termini and lysine side chains were fixed modifications, while methionine oxidation, acetylation of N-termini of proteins, conversion of glutamine to pyro-glutamate and phosphorylation on STY were variable modifications. The false discovery rate was set to 1% for positive identification of proteins and peptides with the help of the reversed mouse Uniprot database in a decoy approach. Copy numbers of EPH/EFN proteins were calculated as described after allocating

the summed MS1 intensities to the different experimental conditions according to their fractional MS3 reporter intensities. See Appendix for further detail.

Supplementary references

1. Potel, C.M., Lin, M.H., Heck, A.J.R. & Lemeer, S. Defeating Major Contaminants in Fe(3+)-Immobilized Metal Ion Affinity Chromatography (IMAC) Phosphopeptide Enrichment. *Mol Cell Proteomics* **17**, 1028-1034 (2018).
2. Kettenbach, A.N. & Gerber, S.A. Rapid and reproducible single-stage phosphopeptide enrichment of complex peptide mixtures: application to general and phosphotyrosine-specific phosphoproteomics experiments. *Anal Chem* **83**, 7635-7644 (2011).
3. Larsen, M.R., Thingholm, T.E., Jensen, O.N., Roepstorff, P. & Jorgensen, T.J. Highly selective enrichment of phosphorylated peptides from peptide mixtures using titanium dioxide microcolumns. *Mol Cell Proteomics* **4**, 873-886 (2005).
4. Raijmakers, R., Kraiczek, K., de Jong, A.P., Mohammed, S. & Heck, A.J. Exploring the human leukocyte phosphoproteome using a microfluidic reversed-phase-TiO2-reversed-phase high-performance liquid chromatography phosphochip coupled to a quadrupole time-of-flight mass spectrometer. *Anal Chem* **82**, 824-832 (2010).
5. Yang, F., Shen, Y., Camp, D.G., 2nd & Smith, R.D. High-pH reversed-phase chromatography with fraction concatenation for 2D proteomic analysis. *Expert Rev Proteomics* **9**, 129-134 (2012).
6. Taus, T. *et al.* Universal and confident phosphorylation site localization using phosphoRS. *J Proteome Res* **10**, 5354-5362 (2011).
