# Supplementary material for: Phosphoproteomics identifies a bimodal EPHA2 receptor switch that promotes embryonic stem cell differentiation
Source: Nat Commun. 2020 Mar 13;11:1357. doi: 10.1038/s41467-020-15173-4 (PMC7070061; doi:10.1038/s41467-020-15173-4)
Supplement: Supplementary file 3 — Reporting Summary [file 41467_2020_15173_MOESM3_ESM.pdf]

nature research

Corresponding author(s): Greg Findlay

Last updated by author(s): 15/01/2020

Reporting Summary

Nature Research wishes to improve the reproducibility of the work that we publish. This form provides structure for consistency and transparency in reporting. For further information on Nature Research policies, see Authors & Referees and the Editorial Policy Checklist .

Statistics

For all statistical analyses, confirm that the following items are present in the figure legend, table legend, main text, or Methods section.

n/a	Confirmed	
☐	☒	The exact sample size (*n*) for each experimental group/condition, given as a discrete number and unit of measurement
☐	☒	A statement on whether measurements were taken from distinct samples or whether the same sample was measured repeatedly
☐	☒	The statistical test(s) used AND whether they are one- or two-sided<br>*Only common tests should be described solely by name; describe more complex techniques in the Methods section.*
☐	☒	A description of all covariates tested
☐	☒	A description of any assumptions or corrections, such as tests of normality and adjustment for multiple comparisons
☐	☒	A full description of the statistical parameters including central tendency (e.g. means) or other basic estimates (e.g. regression coefficient) AND variation (e.g. standard deviation) or associated estimates of uncertainty (e.g. confidence intervals)
☐	☒	For null hypothesis testing, the test statistic (e.g. *F*, *t*, *r*) with confidence intervals, effect sizes, degrees of freedom and *P* value noted<br>*Give P values as exact values whenever suitable.*
☒	☐	For Bayesian analysis, information on the choice of priors and Markov chain Monte Carlo settings
☒	☐	For hierarchical and complex designs, identification of the appropriate level for tests and full reporting of outcomes
☒	☐	Estimates of effect sizes (e.g. Cohen's *d*, Pearson's *r*), indicating how they were calculated

Our web collection on statistics for biologists contains articles on many of the points above.

Software and code

Policy information about availability of computer code

Data collection	Phosphoproteomic data was collected using Thermo Scientific Xcalibur v3.1
Data analysis	Mass spectrometry data was analysed using Thermo Scientific Proteome Discoverer v2.2 and Matrix Mascot search engine.<br>Phosphoproteomic data was analyzed using a standard R package, Limma R v1 and Bioconductor ReactomePA v3.10

For manuscripts utilizing custom algorithms or software that are central to the research but not yet described in published literature, software must be made available to editors/reviewers. We strongly encourage code deposition in a community repository (e.g. GitHub). See the Nature Research guidelines for submitting code & software for further information.

Data

Policy information about availability of data

All manuscripts must include a data availability statement. This statement should provide the following information, where applicable:
- Accession codes, unique identifiers, or web links for publicly available datasets
- A list of figures that have associated raw data
- A description of any restrictions on data availability

Phosphoproteomic profiling data from Fig 1 have been deposited to the ProteomeXchange Consortium via the PRIDE partner repository with the dataset identifier PXD012069. The source data underlying Figs 1e-f; 2a,c-h; 3, 4b-d,f-j; 5a-b,d-e; 6a-d and Supplementary Figs 1a; 2; 3a-d,f-h; 4; 5a-d are provided as a Source Data file. The authors declare that all other data supporting the findings of this study are available within the paper and its supplementary information files.

Field-specific reporting

Please select the one below that is the best fit for your research. If you are not sure, read the appropriate sections before making your selection.

Life sciences study design

All studies must disclose on these points even when the disclosure is negative.

Sample size	No sample size calculations were performed. Experiments were repeated at least 3 times to ensure reproducibility
Data exclusions	No data were excluded
Replication	All attempts at replication were successful
Randomization	Samples were incorporated into experimental analysis at random
Blinding	Investigators analyzed samples in a blind fashion using a code as a sample identifier. Sample identity was then revealed at the end of the analysis

Reporting for specific materials, systems and methods

We require information from authors about some types of materials, experimental systems and methods used in many studies. Here, indicate whether each material, system or method listed is relevant to your study. If you are not sure if a list item applies to your research, read the appropriate section before selecting a response.

Materials & experimental systems

n/a	Involved in the study
☐	☒ Antibodies
☐	☒ Eukaryotic cell lines
☒	☐ Palaeontology
☒	☐ Animals and other organisms
☒	☐ Human research participants
☒	☐ Clinical data

Methods

n/a	Involved in the study
☒	☐ ChIP-seq
☒	☐ Flow cytometry
☒	☐ MRI-based neuroimaging

Antibodies

Antibodies used

ERK1/2 pT202/pY204 Cell Signaling Technology #9101 Lot#28
ERK1/2 Santa Cruz Biotechnology #sc-93 Lot#H2614 (discontinued)
EPHA2 pS898 AbGent #AP3722a-ev Lot#SA091210AB
EPHA2 (N-TERMINAL) MRC Reagents & Services S705D Lot#2
EPHA2 (C-TERMINAL) MRC Reagents & Services S700D Lot#2
pTYR (CLONE 4G10) Millipore #05-321 Lot#2452515
KLF4 R&D Systems #AF3158 Lot#WRR0414021
DNMT3B Imgenex #IMG-184A Lot#AB111609-05 (discontinued)
NANOG Reprocell #RCAB001P Lot#C01MG07
OCT4 Santa Cruz Biotechnology #sc-5279 Lot#C1014
AKT pS473 Cell Signaling Technology #9271 Lot#13
GSK3B pS9 Cell Signaling Technology #9336 Lot# unavailable due to lab closure
CDH1 BD Transduction Laboratories #610881 Lot#3290561
SOX2 Cell Signaling Technology #2748 Lot#4
EFNA1 R&D Systems #AF702 Lot#BWG021606A
STAT3 pY705 Cell Signaling Technology #9131 Lot#30
STAT3α Cell Signaling Technology #8768 Lot#2
SHP2 BD Biosciences #610621 Lot#7341595
α-TUBULIN Sigma-Aldrich T9026 Lot# unavailable due to lab closure
EPHA2 R&D Systems #AF639 Lot#KPC011810A

Validation

ERK1/2 pT202/pY204 Immunoblotting validation data on Cell Signaling Technology website
ERK1/2 Immunoblotting validation data on Santa Cruz Biotechnology website
EPHA2 pS898 Immunoblotting validation data in Figure 1F & on AbGent website
EPHA2 (N-TERMINAL) Immunoblotting validation in Figure 2G
EPHA2 (C-TERMINAL) Immunoblotting validation in Figure 3A
pTYR (CLONE 4G10) Immunoblotting validation data in Figure 3C & on Millipore website
KLF4 Validation data on R&D Systems website & Williams et al, Cell Reports 2016: 16(7): 1820-8
DNMT3B Immunoblotting validation data in Figure S5A & Williams et al, Cell Reports 2016: 16(7): 1820-8
NANOG Immunoblotting validation data in Figure S5B-D

OCT4 Immunoblotting validation data in Figure S5B
AKT pS473 Immunoblotting validation on Cell Signaling Technology website
GSK3B pS9 Immunoblotting validation on Cell Signaling Technology website
CDH1 Validation on BD Transduction Laboratories website
SOX2 Immunoblotting validation data in Figure S5D
EFNA1 Immunoblotting validation data in Figure 3C
STAT3 pY705 Immunoblotting validation on Cell Signaling Technology website
STAT3α Immunoblotting validation on Cell Signaling Technology website
SHP2 Immunoblotting validation on BD Biosciences website
α-TUBULIN Immunoblotting validation on Sigma-Aldrich website
EPHA2 R&D Systems immunostaining validation in Figure S5E

Eukaryotic cell lines

Policy information about cell lines

Cell line source(s)	CCE mouse embryonic cell lines (Kyoto University: https://discovery.lifemapsc.com/stem-cell-differentiation/in-vitro-cells/inner-cell-mass-mus-musculus-cce-kyoto-university) Source: Laboratory of Dr. Janet Rossant, SickKids Research Institute, Toronto, CA
Authentication	Mouse embryonic stem cell lines were routinely authenticated by expression of a panel of embryonic stem cell specific markers including OCT4, NANOG, KLF4 and by alkaline phosphatase staining, which is specific for pluripotent stem cells
Mycoplasma contamination	All cell lines were regularly confirmed negative for mycoplasma contamination throughout the course of this study
Commonly misidentified lines (See ICLAC register)	N/A
